# Flow Cytometry-Based Assessment of Mitophagy Using MitoTracker

**DOI:** 10.3389/fncel.2016.00076

**Published:** 2016-03-30

**Authors:** Bin Xiao, Xiao Deng, Wei Zhou, Eng-King Tan

**Affiliations:** ^1^Department of Neurology, National Neuroscience InstituteSingapore, Singapore; ^2^Department of Neurology, The First Affiliated Hospital, Guangxi Medical UniversityNanning, China; ^3^Department of Neurology, Singapore General HospitalSingapore, Singapore; ^4^Neuroscience Behavioral Disorders Program, Duke-NUS Graduate Medical School, National University of SingaporeSingapore, Singapore

**Keywords:** MitoTracker, mitophagy, reactive oxygen species, mitochondrial potential, neurodegeneration

Mitophagy is an important mechanism in mitochondrial quality control through autophagic clearance of damaged mitochondria and has been considered to assume protective roles against some diseases, especially neurodegeneration, including Parkinson's disease (Haelterman et al., 2014), and Alzheimer's disease (Ye et al., 2015). To understand the role of mitophagy in these diseases, measures to assess mitophagy are of great importance. Among those established methods, engulfment of mitochondria by autophagosome shown by transmission electron microscope (TEM) provides unequivocal evidence of occurrence of mitophagy (Klionsky et al., 2012). However, it demands painstaking effort and is difficult to quantify mitophagy.

MitoTracker has emerged as a useful tool in evaluating mitophagy. Mitochondrial potential-independent MitoTracker is able to stain live mitochondria and enables demonstration of colocalization of mitochondria and autophagosome or lysosome with the aid of corresponding marker (Klionsky et al., 2012). Alternatively, since MitotTracker Green has been used to represent mitochondrial mass (Agnello et al., 2008; Cottet-Rousselle et al., 2011; Zhou et al., 2011) and a decrease in MitotTracker intensity may indicate the degradation of mitochondria, it has been widely utilized to evaluate mitophagy (Kundu et al., 2008; Valentin-Vega et al., 2012). Recently, a flow cytometry-based approach to determine mitophagy by using MitoTracker Deep Red has been introduced by Mauro-Lizcano et al. (2015).

Despite the promising role of MitoTracker, we would like to highlight potential limitations in its interpretation as some factors may significantly affect determination of mitochondrial mass by MitoTracker staining. First, MitoTracker Deep Red is actually a mitochondrial potential-dependent dye though this is usually not highlighted in the manufacturer's manual. It has been used as an index for mitochondrial potential (Lugli et al., 2005; Zhou et al., 2011; Greene et al., 2012). Our data also suggest that the intensity of MitoTracker Deep Red changes along with mitochondrial potential. Mitochondrial depolarization induced by carbonyl cyanide 3-chlorophenylhydrazone (CCCP) treatment decreased fluorescence intensity of MitoTracker Deep Red, while starvation induced by Earle's Balanced Salt Solution (EBSS) treatment increased fluorescence intensity of MitoTracker Deep Red (Figure 1A). JC-1 assay utilized in our experiments confirmed that CCCP disrupted mitochondrial potential and revealed that EBSS treatment caused mitochondrial potential to increase (Figure 1B), in line with the report that amino acid starvation raised mitochondrial potential (Johnson et al., 2014). Our data and previous reports suggest that MitoTracker Deep Red is a mitochondrial potential-dependent dye and therefore should not be used to quantitatively assess mitophagy. MitoTracker Green has been widely utilized to represent the mitochondrial mass (Agnello et al., 2008; Cottet-Rousselle et al., 2011; Zhou et al., 2011). Notwithstanding, we found that EBSS treatment caused increment in the intensity of MitoTracker Green, indicating MitoTracker Green staining might also be affected by mitochondrial potential (Figure 1C). Unexpectedly, our data showed that its intensity increased with CCCP treatment which depolarizes mitochondria (Figure 1C). As CCCP treatment leads to mitochondrial depolarization and reactive oxygen species (ROS) generation, we speculated that both may affect MitoTracker Green staining, with ROS generation induced by CCCP treatment having a more prominent effect on MitoTracker staining than mitochondrial depolarization. The surplus might be responsible for the intensity increase in MitoTracker Green. To test if ROS are able to enhance MitoTracker Green fluorescence, tert-butyl hydroperoxide (TBHP), which is organic peroxide, was applied to the cells. Indeed, it resulted in modest but significant increment of MitoTracker Green fluorescence (Figure 1C). Although ROS have been reported to affect mitochondrial biogenesis, the result from our western blot experiments did not reveal any changes in the proteins from different compartments of mitochondria upon various treatments, including mitophagy inducer CCCP or nicotinamide (NAM), ROS inducer TBHP and autophagy inducer EBSS (Figure 1D). Moreover, N-acetyl-L-cysteine (NAC), a ROS scavenger, was able to reverse CCCP or TBHP-induced changes in the intensity of MitoTracker Green, suggesting that the changes caused by CCCP or TBHP are ROS-related. NAC failed to suppress EBSS induced intensity increase which may be caused mainly by enhanced mitochondrial potential (Figure 1E). The process of MitoTracker dye specific labeling mitochondria consists of its enrichment in mitochondria and its interaction with mitochondria. MitoTracker probes passively diffuse across the plasma membrane and then accumulate in active mitochondria in a potential-dependent manner. Subsequently, it covalently binds the thiol groups of the cysteine residues of mitochondrial proteins (Chazotte, 2011). It has been claimed that MitoTracker stains mitochondria independent of mitochondrial potential after its covalent binding to mitochondria. However, mitophagy inducers, such as CCCP treatment, usually have to be applied for a relatively long period to trigger mitophagy. In addition, the activity of cysteine containing proteins in mitochondria may be affected by covalent association of MitoTracker to the thiol groups. Hence, MitoTracker incubation for analysis is usually performed after mitophagy-related treatments (Mauro-Lizcano et al., 2015). This procedure makes MitoTracker staining of mitochondria dependent of mitochondrial potential, as the mitophagy-related treatments usually interfere with mitochondrial potential, impeding MitoTracker accumulation in mitochondria. Our abovementioned data garnered with MitoTracker incubation after drug treatment demonstrated a close correlation between MitoTracker fluorescence and mitochondrial potential. This might be due to the fact that mitochondrial potential greatly influences enrichment of MitoTracker in mitochondria.

**Figure 1:**
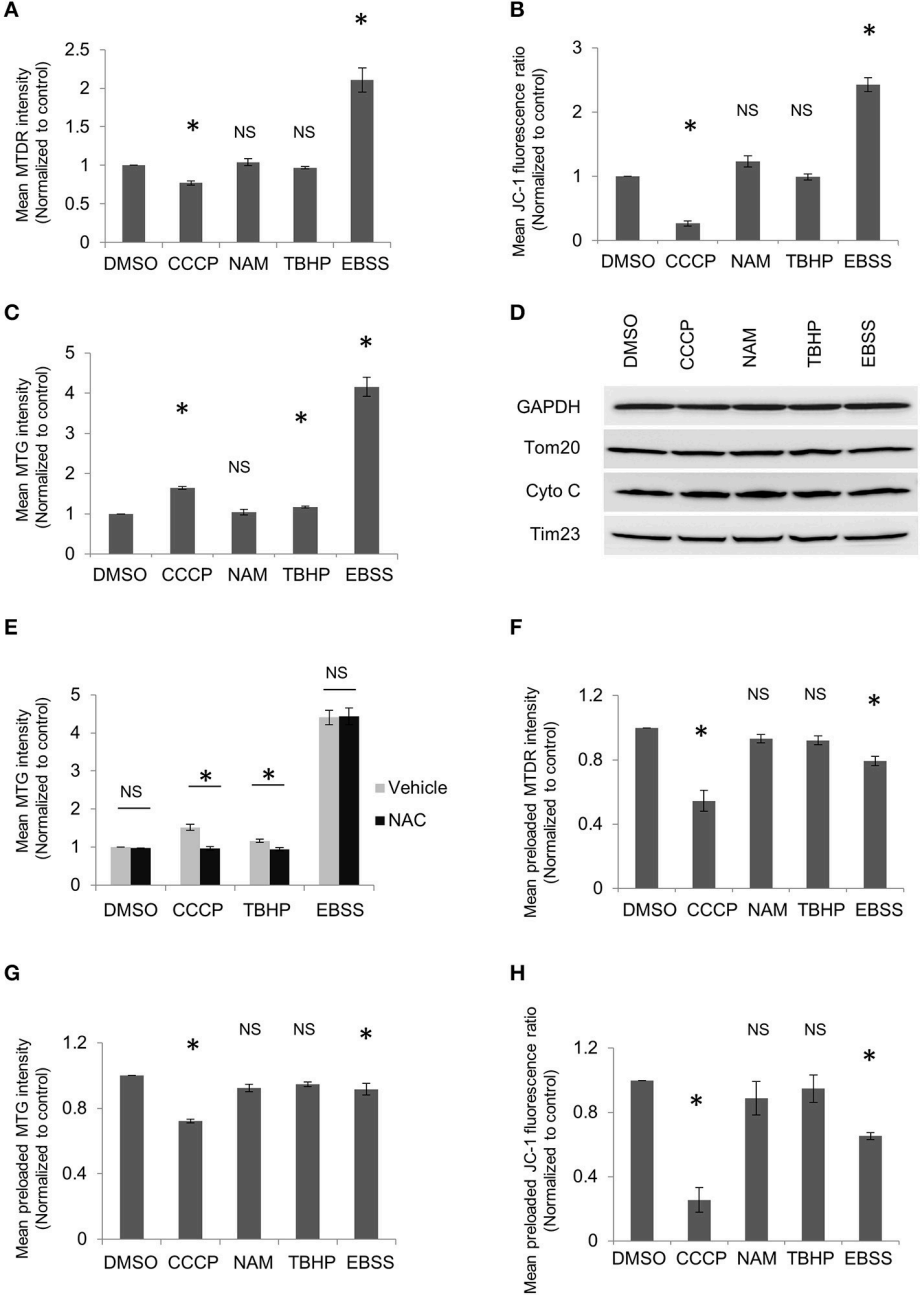
**MitoTracker dyes are affected by mitochondrial potential or ROS**. HeLa cells were treated with DMSO, 10 μM CCCP, 5 mM NAM, or 20 μM TBHP for 2 h or EBSS for 1 h. Cells were harvested and stained with MitoTracker Deep Red (MTDR) **(A)**, JC-1**(B)**, or MitoTracker Green (MTG) **(C)**, followed by flow cytometric analysis. The ratio of the red to green fluorescence intensity in JC-1 stained cells was used to represent mitochondrial membrane potential. **(D)** HeLa cells were treated as in **(A)** and followed by western blot to examine the levels of mitochondrial proteins from different compartments. **(E)** HeLa cells were treated with DMSO, CCCP, or TBHP along with or without 1 mM NAC for 2 h or EBSS with or without 1 mM NAC for 1 h. Cells were then collected and incubated with MTG for flow cytometric analysis. HeLa cells were incubated with MTDR **(F)**, MTG **(G)**, or JC-1 **(H)** for 20 min, washed and then followed by treatment as in **(A)**. Cells were collected and analyzed using flow cytometry. The error bars represent SEM from three independent experiments; at least 10,000 cells were analyzed per experiment. Statistical significance was assessed by two-tailed Student's *t*-test. Asterisks denote a statistically significant difference from control (*p* < 0.05); NS, not significant (*p* > 0.05).

To further elucidate if mitochondrion-bound MitoTracker dyes could retain in mitochondria regardless of mitochondrial potential, experiments were also carried out with MitoTracker loaded prior to drug treatments. We found that CCCP treatment reduced fluorescence intensity in both MitoTracker Deep Red and MitoTracker Green (Figures 1F,G), rather than enhancing MitoTracker Green fluorescence when the dye was loaded after drug treatment. Moreover, EBSS treatment also decreased MitoTracker intensity if the dyes were loaded after treatment. We reason that the washout of MitoTracker after preincubation invalidates the capacity of mitochondria to incorporate free dye. Consistently, mitochondrial potential-dependent dye JC-1 revealed decrease in mitochondrial potential if the dye was preloaded and washed prior to EBSS treatment (Figure 1H). Hence, the washout step after preincubation of MitoTracker makes it impossible to detect mitochondrial proliferation, which constitutes an integral part of dynamic mitochondrial biogenesis. Moreover, MitoTracker Green fluorescence diminished upon CCCP or EBSS treatment if it was preloaded (Figure 1G), sharing a similar trend as changes in preloaded JC-1 (Figure 1H). This indicates that mitochondrion-bound MitoTracker Green is not linked to mitochondria as tightly as claimed and may be released off mitochondria like other mitochondrial potential-dependent dye. Together, our data indicate that MitoTracker dyes, including MitoTracker Green, are mitochondrial potential-dependent, and may be affected by ROS level in cells as well.

Changes in the fluorescence intensity of MitoTracker dyes can be caused by mitochondrial protein degradation mediated by mitochondria-derived vesicles (McLelland et al., 2014), proteasome (Yoshii et al., 2011), or mitochondrial proteases (Klionsky et al., 2012) in addition to autophagic clearance of mitochondria. This nonspecificity has been well addressed by the addition of autophagy inhibitors (Kundu et al., 2008; Mauro-Lizcano et al., 2015). If autophagy inhibitor is able to reverse the alterations in MitoTracker, the conclusion that mitophagy is responsible for the changes should be plausible. More importantly, a mitochondrial dye utilized to assess mitophagy entails its specific representing the mitochondrial mass. In our study, we utilized HeLa cells which have no detectable endogenous Parkin, an important component for mitophagy. Therefore, one advantage HeLa cells have over other cell lines is that drug treatment-induced MitoTracker changes in these cells will likely be caused by other factors than mitophagy, though we cannot exclude Parkin-independent mitophagy. In addition, all the drug treatment lasted for only 2 h, precluding the possibility of significant mitochondrial biogenesis. Together with the western blot results, it is convincing that no significant mitophagy-involved gain or loss of mitochondrial mass occurred in our study. Instead, the changes in MitoTracker Green or MitoTracker Deep Red in response to CCCP, EBSS, or TBHP are related to shift in mitochondrial potential or ROS outburst, though they are not as pertinent as those detected by the dyes specifically designed for these aspects, such as JC-1 or dihydroethidium. Since mitochondrial depolarization and ROS are intricately implicated in mitophagy, any assay which may be affected by mitochondrial depolarization or ROS has its intrinsic defect in evaluating mitophagy. Also, it has been shown that CCCP may cause redistribution of mitochondrial dyes, including MTR and MTG, from mitochondria to the cytosol, and subsequently to lysosome, which is irrelevant to the engulfment of mitochondria by lysosome (Padman et al., 2013). It is therefore important to elucidate if other mitophagy inducers also have similar effect on the mitochondrial dyes before any such dye is employed to evaluate mitophagy.

In essence, our data clearly show that currently used mitochondrial dyes, including MitoTracker Deep Red or MitoTracker Green, may be affected by mitochondrial potential or ROS. Taking these limitations into account, we suggest that quantitative assessment of mitophagy using MitoTracker should be always interpreted along with other mitophagy assays. Meanwhile, we await future development of a more specific marker for precise measurements of mitophagy.

## Author contributions

BX and ET conceived and designed the experiments. BX, XD, and WZ performed the experiments. BX and ET analyzed the data and wrote the paper.

### Conflict of interest statement

The authors declare that the research was conducted in the absence of any commercial or financial relationships that could be construed as a potential conflict of interest.
